# Multi-Joint Bionic Mechanism Based on Non-Circular Gear Drive

**DOI:** 10.3390/biomimetics8030272

**Published:** 2023-06-27

**Authors:** Dawei Liu, Tao Zhang, Yuetong Cao

**Affiliations:** 1 College of Mechanical Engineering, Yanshan University, Qinhuangdao 066004, China; 2 National Engineering Technology Research Center of Cold Rolling Strip Equipment and Technology, Yanshan University, Qinhuangdao 066004, China

**Keywords:** multi-joint bionic mechanism, cable drive mechanism, non-circular gear, motion decoupling

## Abstract

Aiming at the nonlinear expansion/contraction drive problem between different cables in multi-joint cable drive mechanisms, a mechanical drive method based on a non-circular gear drive was proposed, which could replace the servo-sensing control system and minimize the system’s complexity and cost. A multi-joint single-degree-of-freedom (DOF) bending mechanism was constructed with several T-shaped components and cross-shaped components. The principle of the multi-joint mechanism driven by non-circular gears was clarified. The corresponding relationships between the joint bending angle, cables’ extension/retraction amount and non-circular gear transmission ratio were established. Using the Bowden cable driving, a multi-DOF bending mechanism decoupling scheme was proposed. Considering the adverse effect of non-circular gear hysteresis on the motion of multi-joint mechanisms, a non-circular gear backlash elimination method was proposed. The expression of the backlash of the non-circular gear with respect to the axial movement amount was deduced, which could enable the precise control of the backlash. A two-DOF multi-joint bionic mechanism driven by the non-circular gear was developed. The experimental results show that the mechanism can achieve coordinated bending motion by precisely controlling the line extension/contraction through non-circular gears. This multi-joint bionic mechanism driven by non-circular gears has the characteristics of reliable structure and simple control, and it is expected to be applied to bionic fish and bionic quadruped robots in the future.

## 1. Introduction

The spine is an essential feature of higher animals. The functions of the spine include supporting the trunk, storing and transmitting energy, improving energy utilization, and ensuring flexibility and stability. Mechanical bionic spines have essential applications in bionic robots such as quadruped robots [1], underwater robotic fish [2,3], flexible manipulators [4], etc.

Studies have been conducted in China and abroad on bionic spine mechanisms. Currently, their driving methods can be classified into two categories: built-in pneumatic actuation, and external cable-driven actuation. The built-in pneumatic actuation means that the drive is built-in. The bending and extension of each section are achieved by controlling the input air pressure in the pneumatic actuator. Using octopus tentacles, elephant trunks, and human arms as inspiration, the German manufacturer of Festo automation technology has launched a gas-powered bionic robot [5] that enables the wrapping and grasping of objects. In 2021, Prof. Jingtao Lei’s team at Shanghai University proposed a pneumatic muscle-driven rigid–flexible coupling bionic body [6,7] that can achieve variable stiffness in lateral bending. Moreover, a series–parallel stiffness model of a bionic body was proposed, and its active variable stiffness characteristics were analyzed. The experiments showed that the mechanism could achieve dynamic bending of the bionic body, which can improve the flexibility and impact resistance of the bionic leg. The soft-body robots proposed in the literature [8,9] are all based on the prototype of a structure with a flexible fiber outer tube wrapped with a driving gas, which enables the soft-body robot to achieve various motions by inputting different gas pressures. Liu et al. [10] proposed a collapsible pneumatic soft manipulator that can achieve finger-like extension and contraction functions. At the same time, the mechanical structure was introduced, and the kinematic model of the pneumatic module was established. The above bionic spine mechanisms all adopt the built-in gas-driven scheme. Although gas has good variable stiffness characteristics, due to the large compressibility of gas, motion control by controlling the input of air pressure will limit the control precision of the mechanical body. At the same time, it is also a big problem that the driving device is built-in, resulting in an excessively large volume and bloated structure of the mechanism.

Compared with the built-in pneumatic actuation mechanism, the external cable drive dramatically reduces the joint’s weight and rotational inertia and improves its transmission ratio by separating the machine body from the drive. At the same time, compared with gas, the cord has better control and higher stability. Thus, domestic and foreign scholars have also conducted much research on cord-driven bionic spines. The surgical manipulator mentioned in the literature [11] uses a spring and a circular tube to form a flexible joint. Three ropes are distributed around the circular tube, and the bending motion of the flexible joint is achieved by controlling the change in the expansion and contraction of the ropes. It has been verified through tests that the manipulator has good stability and accuracy. Suh J et al. [12,13] proposed a surgical robot with a non-elastic pulley rolling joint to solve the problem of a small angular bending radius that is difficult to achieve with conventional flexible joints consisting of circular tubes. The surgical robot is suitable for scenarios such as low tension on the drive cord and a small bending radius of curvature. However, this surgical robot has no internal supporting elements, making it unstable when it is static. Based on this, the bionic mechanism proposed in this paper adds an elastic element, which improves the machine’s stability. Geng Hao et al. [14] used the telescopic and connecting rods as the robotic arm parts. Meanwhile, the corresponding cord was driven by five motors to control the deflection of the connecting rod. Wei [15,16] designed a linear-driven continuous manipulator with 6 degrees of freedom and a 1.5 kg load. The kinematic mapping methods of drive space, operation space, and joint space of the single-stage flexible joint were analyzed. Then, the decoupling kinematic algorithm of the three-stage flexible joint was given. The literature [17,18] solely concerns wire-driven robots. The robot consists of an external rigid frame and an end effector. In addition, the experimental validations all show the universal applicability and great motion potential of external wire-driven bionic robots.

The external cable drive mechanism offers better control accuracy and stability than the internal gas drive mechanism. However, the amount of change in the elongation and shortening of the cable during traction is nonlinear, so the existing cable-driven multi-joint mechanism of the bending module requires at least two cables for traction control. In order to achieve the coordinated motion of nonlinear extension and contraction of the cable, a motor is needed to drive the cable. Moreover, a sensor system must be used in the mechanism so that multiple sensors, multiple servo motors, and multiple harnesses can work together to form a fully closed-loop servo control. The need for a large number of motors, the expensive production cost, and the complicated control process have become urgent technical problems to be solved for the cable-driven multi-joint mechanism. Therefore, this paper proposes a bionic multi-joint mechanism based on nonlinear transmission characteristics of non-circular gears, which can achieve multi-joint bending control without a servo-sensing system. A variable-thickness non-circular gear-gap elimination method is proposed for high-precision joint mechanisms. The feasibility of non-circular gear bionic multi-joint mechanisms is verified by prototype tests, which can provide simple control and fast response of underdrive multi-joint mechanisms for various bionic robots.

## 2. Principle of the Single-DOF Bending Mechanism

### 2.1. Single-DOF Simplest Bending Mechanism

**Figure 1:**
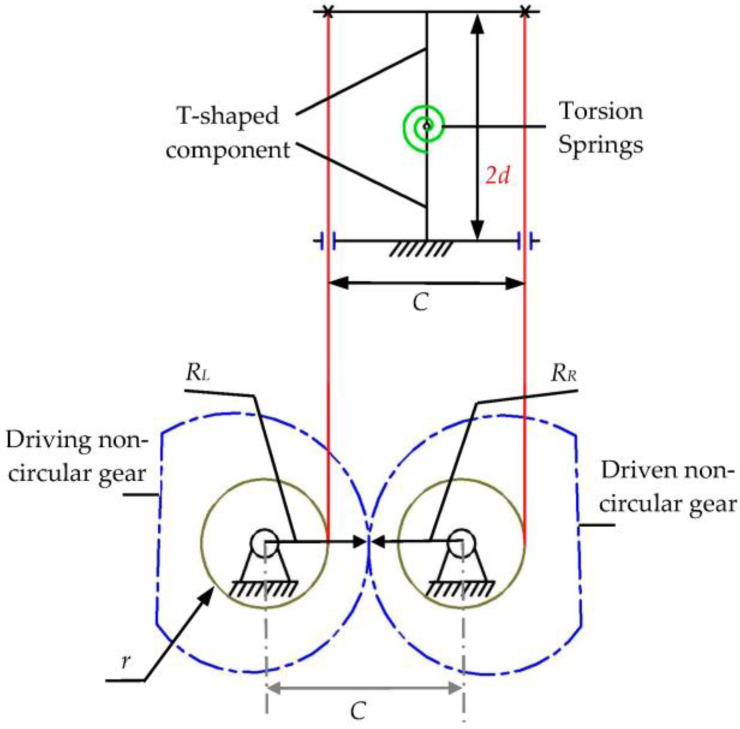
Schematic diagram of the simplest bending mechanism.

Figure 1 shows a single-DOF simplest bending mechanism consisting of a set of bending elements and a motion control mechanism. The bending unit consists of two hinged T-shaped components and a torsional spring. The motion control mechanism consists of a pile of non-circular gears, two cables, and two cable wheels. A T-shaped component in the bending unit is fixed to the base. The torsion spring can assist the movable T-shaped component in returning to its initial position (the vertical position of the movable T-shaped component in Figure 1). One end of the two strings is fixed on the movable T-shaped component, and the other end is wound on the cable wheel through a hole in the fixed T-shaped component. Two cable wheels are fixedly connected to two non-circular gears. Based on the nonlinear transmission characteristics of the non-circular gear, the extension and shortening rules of the two cables are controlled. Then, the T-shaped components can be driven to swing from side to side. The design of the non-circular gear is the key to realizing the motion control of the movable T-shaped components.

**Figure 2:**
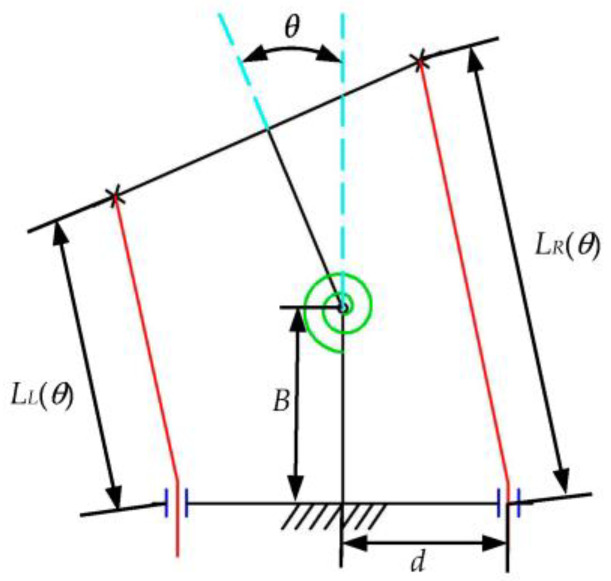
Schematic diagram of the simplest bending element joint.

In Figure 1, *C* is the center distance of the non-circular gear (mm); *r* is the radius of the cable wheel, and both wheels have the same radius (mm); *R_L_* and *R_R_* are the directional diameters of the driving and driven non-circular gears (mm), respectively. The main parameters of the two T-shaped components in the simplest bending mechanism are given in Figure 2. The structural parameters of the two T-shaped components are the same. *B* is the length (mm) of the T-shaped component; 2d is the width (mm); *θ* is the rotation angle (rad) of the upper movable T-shaped component relative to the lower fixed T-shaped component; *L_L_*(*θ*), *L_R_*(*θ*) indicates the distance (mm) between the left and right sides of the line cord between the two T-shaped components when the rotation angle of the T-shaped component is *θ*. According to the geometric relationship:

(1) $$\left\{\begin{array}{l} L_{L}(\theta )=2B\cos\frac{\theta }{2}-2d\sin\frac{\theta }{2}\\ L_{R}(\theta )=2B\cos\frac{\theta }{2}+2d\sin\frac{\theta }{2} \end{array}\right.$$

When the movable T-shaped component is in the initial position *θ* = 0, the cable length on both sides is as shown by Equation (2):

(2) $$L_{L}(0)=L_{R}(0)=2B$$

In combining Equations (1) and (2), it can be seen that when the bending mechanism moves and the T-shaped component turns *θ* counterclockwise, the changes Δ*L_L_* and Δ*L_R_* of the extension and contraction of the cable on the left and right sides, respectively, are as shown by Equation (3):

(3) $$\left\{\begin{array}{l} \Delta L_{R}=L_{R}(\theta )-L_{R}(0)=2B(\cos\frac{\theta }{2}-1)+2d\sin\frac{\theta }{2}\\ \Delta L_{L}=L_{L}(0)-L_{L}(\theta )=2B(1-\cos\frac{\theta }{2})+2d\sin\frac{\theta }{2} \end{array}\right.$$

Two cables are wound on the cable wheels and driven by the wheel, which results in changes in extension and contraction. Thus, the following relationship between the strings and the spool is shown in Equation (4):

(4) $$\left\{\begin{array}{l} \Delta L_{L}=r\theta_{L}\\ \Delta L_{R}=r\theta_{R} \end{array}\right.$$

In Equation (4), *θ_L_* and *θ_R_* are the rotation angles of the left and right cable-driven wheels, respectively, when the movable T-shaped component turns *θ*. Because the cable wheel and the driving and driven non-circular gears are solidly connected, *θ_L_* and *θ_R_* are the rotation angles of the driving and driven non-circular gears, respectively. Significantly, the transmission ratio of the non-circular gears is equal to the transmission ratio of the cable wheel. According to Equations (3) and (4), the transmission ratio function of the non-circular gears can be obtained as follows:

(5) $$i_{12}=\frac{\omega_{1}}{\omega_{2}}=\frac{d\theta_{L}}{d\theta_{R}}=\frac{B\sin\frac{\theta }{2}+d\cos\frac{\theta }{2}}{B\sin\frac{\theta }{2}-d\cos\frac{\theta }{2}}$$

where *ω*_1_ and *ω*_2_ are the angular velocities (rad/s) of the two wire wheels. Ignoring the thickness of the cable wound on the cable wheel, the distance *C* between the two non-circular gear shafts is constant, and *R_R_* + *R_L_* = *C*. Based on Equations (4) and (5), the pitch curve equation of the driving non-circular gear can be obtained as follows:

(6) $$\left\{\begin{array}{l} R_{L}=C\frac{d\cos\frac{\theta }{2}+B\sin\frac{\theta }{2}}{2d\cos\frac{\theta }{2}}\\ \theta_{L}=\frac{2}{r}\left[2B(1-\cos\frac{\theta }{2})+2d\sin\frac{\theta }{2}\right] \end{array}\right.$$

The equation of the pitch curve of the driven non-circular gear is Equation (7):

(7) $$\left\{\begin{array}{l} R_{R}=C\frac{d\cos\frac{\theta }{2}-B\sin\frac{\theta }{2}}{2d\cos\frac{\theta }{2}}\\ \theta_{R}=\frac{2}{r}\left[2B(1-\cos\frac{\theta }{2})-2d\sin\frac{\theta }{2}\right] \end{array}\right.$$

### 2.2. Single-DOF Multi-Joint Bending Mechanism

**Figure 3:**
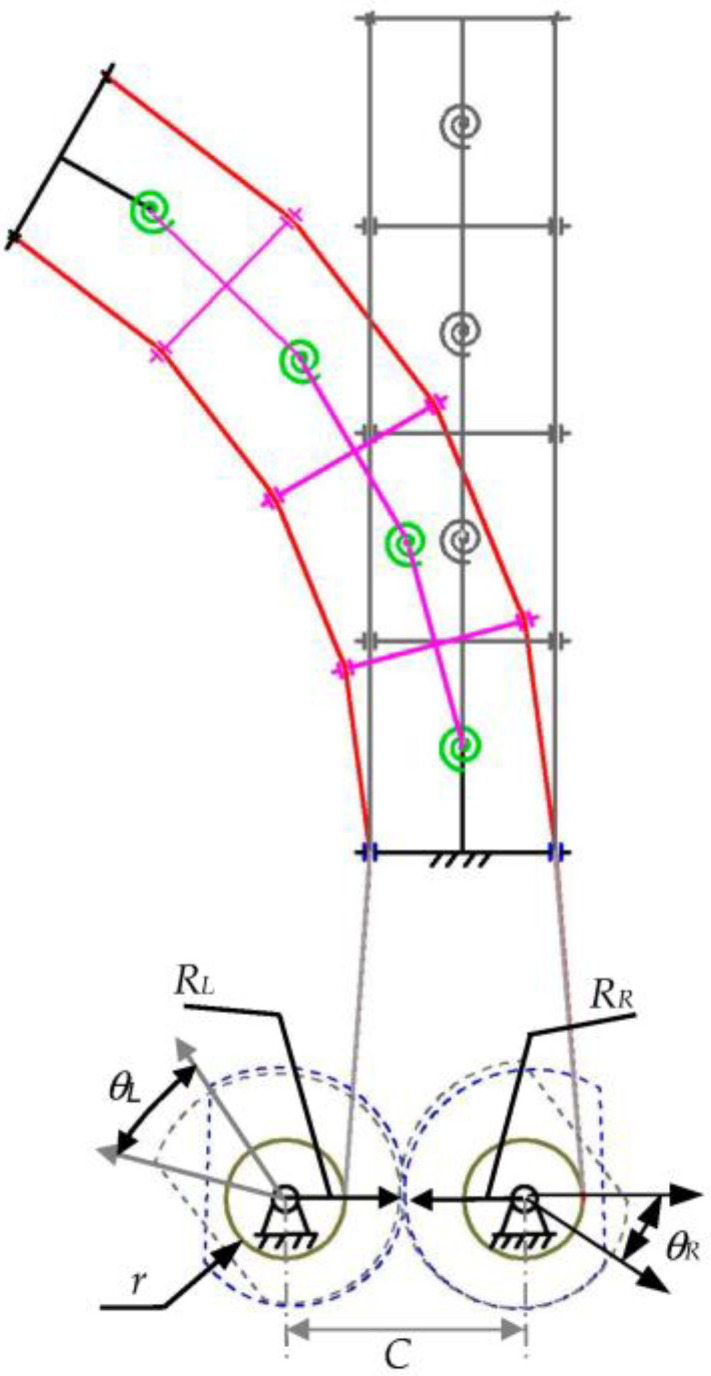
Single-DOF multi-joint bending mechanism.

Based on the single-DOF simplest bending mechanism, the cross-shaped component is added to obtain the single-DOF multi-joint bending mechanism, as shown in Figure 3. On the basis of the first and last two T-shaped components, a number of hinged cross-shaped components are added. Meanwhile, a torsion spring at every two hinged components can help the components return to their initial position. One end of the two strings is fixed on the movable T-shaped component, which passes through the holes at both ends of each component in turn, before finally being wound on the wire wheel. The mechanism can achieve the function of sizeable overall bending through slight rotation of local joints, similar to an animal’s spine. Thus, it is called a single-DOF bionic mechanism.

The motion of the single-DOF bionic mechanism in Figure 3 is driven by a pair of non-circular gears, two cables, and two cable wheels. With the increase in the number of components, the corresponding relationship between the extension and contraction of the two cables and the joint angle changes. In Figure 3, ignoring the friction between the cable and the joint and joint hinge, according to the assumption of piecewise constant curvature (PCC) [19,20], it can be considered that the rotation angle between the joints is equal.

The total number of T-shaped components and cross-shaped components is *N* + 1. Then, the overall bending angle of the mechanism is *Φ* = *Nθ*. It can be seen from the above section that when *θ* is rotated, the variation of the cable driving length between a single joint is Δ*L_R_* and Δ*L_L_*. Equation (4) can be used to deduce that the variation of the overall cable driving length of the bending module has the following relationship with the driving angle of the cable wheel:

(8) $$\left\{\begin{array}{l} N\Delta L_{L}=r\theta_{L}\\ N\Delta L_{R}=r\theta_{R} \end{array}\right.$$

In Section 2.1, the detailed derivation process of the ratio function and the pitch curve equation of the non-circular gear are given. Similarly, the pitch curve equations of the driving and driven non-circular gears in the single-DOF multi-joint bending mechanism can be obtained. The pitch curve equation of the driving non-circular gear is Equation (9):

(9) $$\left\{\begin{array}{l} R_{L}=C\frac{d\cos\frac{\theta }{2}+B\sin\frac{\theta }{2}}{2d\cos\frac{\theta }{2}}\\ \theta_{L}=\frac{N}{r}\left[2B(1-\cos\frac{\theta }{2})+2d\sin\frac{\theta }{2}\right] \end{array}\right.$$

The equation of the pitch curve of the driven non-circular gear is Equation (10):

(10) $$\left\{\begin{array}{l} R_{R}=C\frac{d\cos\frac{\theta }{2}-B\sin\frac{\theta }{2}}{2d\cos\frac{\theta }{2}}\\ \theta_{R}=\frac{N}{r}\left[2B(1-\cos\frac{\theta }{2})-2d\sin\frac{\theta }{2}\right] \end{array}\right.$$

## 3. Multi-DOF Multi-Joint Bending Mechanism

**Figure 4:**
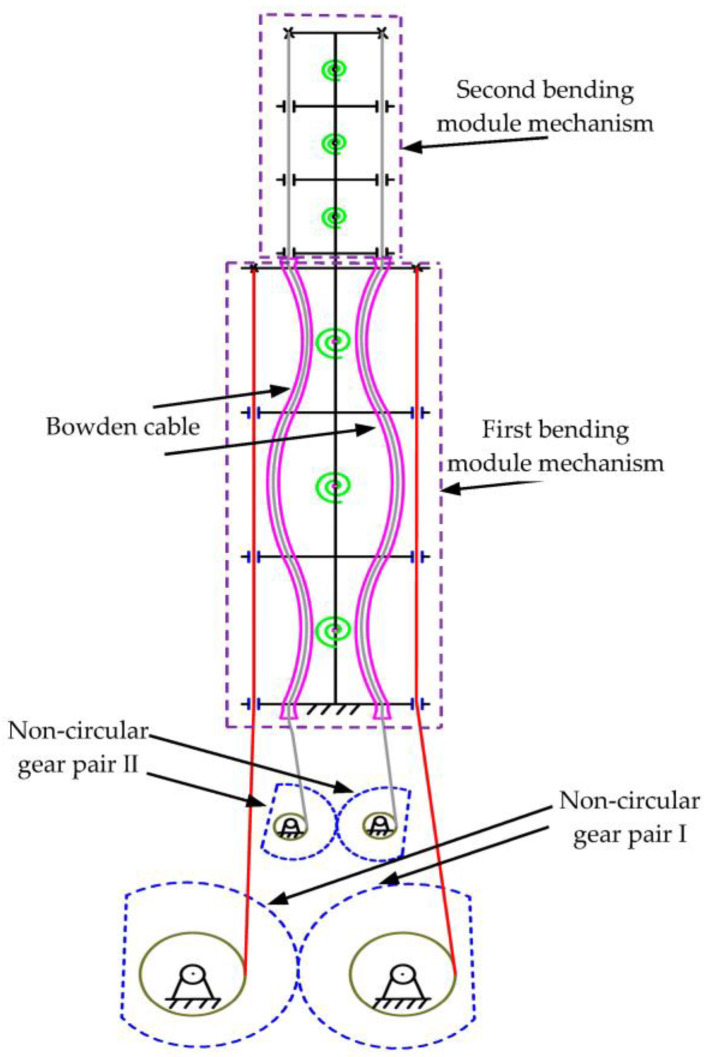
Diagram of the two-DOF bending mechanism.

As shown in Figure 4, based on the single-DOF multi-joint bending mechanism, a second bending module mechanism, two Bowden cables, and a non-circular gear pair II were added to the first T-shaped component to form a two-DOF multi-joint bionic mechanism. According to the design requirements, the above operation scheme was repeated to establish the multi-DOF multi-joint bending mechanism based on the two-DOF multi-joint bending mechanism.

Non-circular gear pairs I and II drive the first and second bending modules, respectively. The first T-shaped component of the first bending module mechanism is fixedly connected with the end T-shaped component of the second bending module mechanism.

When the first module mechanism bends under the traction of the cable, the second module mechanism will carry out the accompanying motion, which leads to the change of the driving cable of the second module mechanism, resulting in the joint coupling problem. In order to solve the joint coupling problem caused by the motion of the two-DOF mechanism, the Bowden cable was selected as the driving cable of the second bending module.

The Bowden cable comprises two parts: a line pipe and a Bowden line rope. The line pipe is wrapped around the Bowden line rope and can slide freely along the Bowden line rope. The Bowden line rope and the line pipe can be bent to a limited degree without changing their length. The Bowden cable’s two ends are fixed at the end of the second bending module mechanism’s rope hole outlet and the front end of non-circular gear pair II. When the machine is stationary, the Bowden cable is in a relaxed state. As the movement of the first bending module leads to additional movement of the second bending module, the Bowden line tube begins to stretch in the relaxed bending state. At this time, the cable used to drive the second bending module has no change in extension or shortening, which means that the joints of the second bending module do not produce bending motion. This method can solve the joint coupling problem caused by the multi-DOF motion of the bionic mechanism.

The mechanism can be controlled by two non-circular gear pairs to make it move in two DOF in a coordinated manner, which is more suitable for animal spine models.

## 4. Eliminate Backlash for Beveloid Non-Circular Gears

The critical problem to achieve the bionic mechanism’s motion is the design of non-circular gears. In order to achieve the cycling and reciprocating swing of the bionic mechanism in its range of motion, the non-circular gear must be able to move forward and in reverse. Due to the presence of backlash, the rotation error will increase and the transmission accuracy will decrease when the non-circular gear moves forward and backward. Because of the high precision requirement of this mechanism, it is essential to eliminate the backlash between non-circular gear pairs.

### 4.1. Eliminate Backlash Principle of Parallel-Shaft Beveloid Non-Circular Gears

The geometric feature of the beveloid gear is that the modification coefficient on each end section of the tooth profile changes linearly along the axis. Because the modification coefficient of the tooth profile of each end section is different, the shape of the involute is slightly different. The overall appearance of the tooth shape is conical. The end of the positive coefficient is called the “big end”, and the end of the negative coefficient is called the “small end”. The tooth thickness of one gear on the pitch circle is equal to the space width of the other gear, in order to make the backlash of a pair of gears be zero during meshing transmission.

Therefore, before meshing transmission of the parallel-shaft beveloid gear pair, by adjusting its axial installation position, the big end of one gear’s meshing corresponds to the small end of the other gear to eliminate backlash.

### 4.2. Enveloping Principle of Parallel-Shaft Beveloid Non-Circular Gears

**Figure 5:**
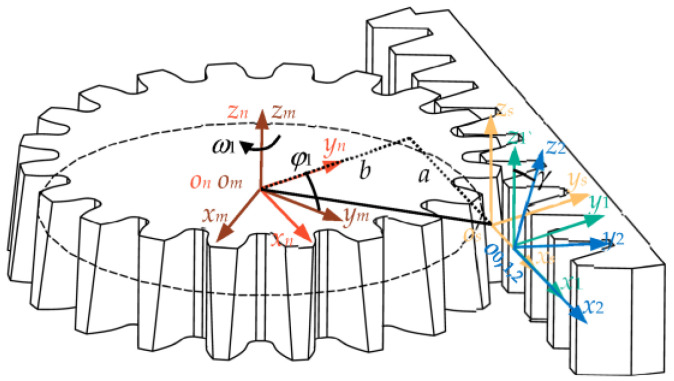
Coordinate axis for machining a beveloid non-circular gear.

For beveloid gears, the formation principle is to tilt the dividing surface of the rack to a certain angle relative to the gear so that the rack’s pitch plane and the gear’s pitch cylinder surface always maintain a pure rolling motion relationship. The formation principle of beveloid gears is extended to the field of variable transmission ratio of non-circular gears, and beveloid non-circular gears can be obtained. When cutting teeth for beveloid non-circular gears, the rack’s dividing surface is tilted at a certain angle so that the rack’s pitch curve and the non-circular gear’s pitch curve always remain purely rolling. Figure 5 shows the machining principle of a beveloid non-circular gear.

**Figure 6:**
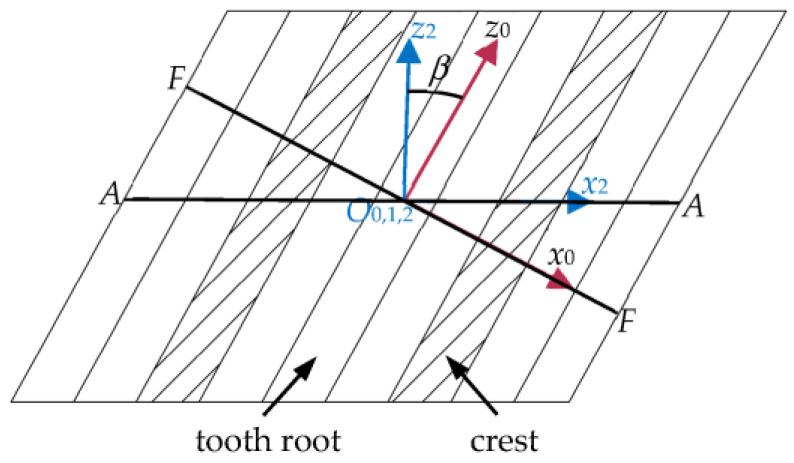
Schematic diagram of the regular section of the rack.

In Figure 5, *S_n_*(*O_n_*-*x_n_y_n_z_n_*) is a fixed coordinate axis, where *x_n_O_n_y_n_* is coplanar with the middle plane of the rack’s face width; *S_m_*(*O_m_*-*x_m_y_m_z_m_*) is the dynamic coordinate axis that rotates with the non-circular gear. The non-circular gear has the *z_m_* axis as the rotation axis. The angular velocity is *ω*_1_, and the rotation angle is *φ*_1_. *S_s_* (*O_s_*-*x_s_y_s_z_s_*) is a moving coordinate axis based on the tangent point *O_s_* of non-circular gears and racks. The coordinate axes *x_s_*, *y_s_*, *z_s_* and *x_n_*, *y_n_*, *z_n_* are parallel. *S*_0_(*O*_0_-*x*_0_*y*_0_*z*_0_), *S*_1_(*O*_1_-*x*_1_*y*_1_*z*_1_), and *S*_2_(*O*_2_-*x*_2_*y*_2_*z*_2_) are the floating coordinate axes connected with the rack. The origins *O*_0_, *O*_1_, and *O*_2_ are located at the midpoint of the rack’s indexing line. The *x*_0_*O*_0_*y*_0_ plane is coplanar with the regular section of the diagonal rack. Section *F*-*F* in Figure 6 is the regular section of the rack. The angle between the *z*_0_ and *z*_2_ axes is the helix angle *β* of the diagonal rack. In the *S*_1_ coordinate system, *x*_1_*O*_1_*y*_1_ is the pitch plane of the rack. The angle between the *z*_1_ axis and the *z*_2_ axis is the rack roll angle *γ*.

When the non-circular gear rotates at a certain angle, the rack will be shifted by a corresponding distance. At the same time, the cut points *O_s_* and *O*_1_ will no longer coincide. *S* is the horizontal movement distance of the rack. The distance between the cut points *O_s_* and *y_n_* is *a*, and the distance between the cut points *O_s_* and *x_n_* is *b*.

Referring to reference [20], the parameter expression of beveloid non-circular gears is finally obtained as follows:

(11) $$\left\{\begin{array}{l} x_{1}=\left(A+G\sin\varphi_{1}\right)x_{3}-Ey_{3}+(C-D\sin\gamma )z_{3}\\ \;+(a+S)\cos\varphi_{1}-b\sin\varphi_{1}\\ y_{1}=\left(D-C\sin\gamma \right)x_{3}+Fy_{3}+(B+A\sin\delta )z_{3}\\ \;+(a+S)\sin\varphi_{1}+b\cos\varphi_{1}\\ z_{1}=-\cos\gamma \sin\beta -\sin\gamma y_{3}+\cos\gamma \cos\beta z_{3} \end{array}\right.$$

where A = cos*β*cos*φ*_1_, B = sin*β*sin*φ*_1_, C = sin*β*cos*φ*_1_, D = cos*β*sin*φ*_1_, E = cos*γ*sin*φ*_1_, F = cos*γ*cos*φ*_1_, and G = sin*β*sin*γ*.

### 4.3. Parallel-Shaft Beveloid Non-Circular Gear Geometric Parameter Solution

Reference [20] shows that when drawing the tooth shape of a non-circular gear, the number of reduced teeth is the number of teeth of a circular gear needed to represent the tooth shape of a non-circular gear. The formula of converted teeth is Equation (12):

(12) $$z=\frac{2\rho }{m_{t}}$$

where *m_t_* is the transverse modulus of the parallel-shaft beveloid non-circular gear and *ρ* is the curvature radius of a non-circular gear. The radius of curvature is derived using Equation (13):

(13) $$\rho =\frac{{\left[R^{2}+{\left(\frac{dR}{d\theta }\right)}^{2}\right]}^{\frac{3}{2}}}{R^{2}+2{\left(\frac{dR}{d\theta }\right)}^{2}-R\frac{d^{2}R}{d\theta^{2}}}$$

where *R* is the radial vector of the non-circular gear and *θ* is the polar angle of the non-circular gear.

When machining beveloid non-circular gears, the dividing surface of the rack cutter is inclined to a certain angle relative to the axis of the gear cutter. Then, the transverse modulus and normal modulus of parallel-shaft beveloid non-circular gears will no longer be equal. It can be seen from [21] that there is the following relationship between the transverse modulus *m_t_* and the normal modulus *m_n_*:

(14) $$m_{t}=\frac{m_{n}}{\cos\beta }$$

**Figure 7:**
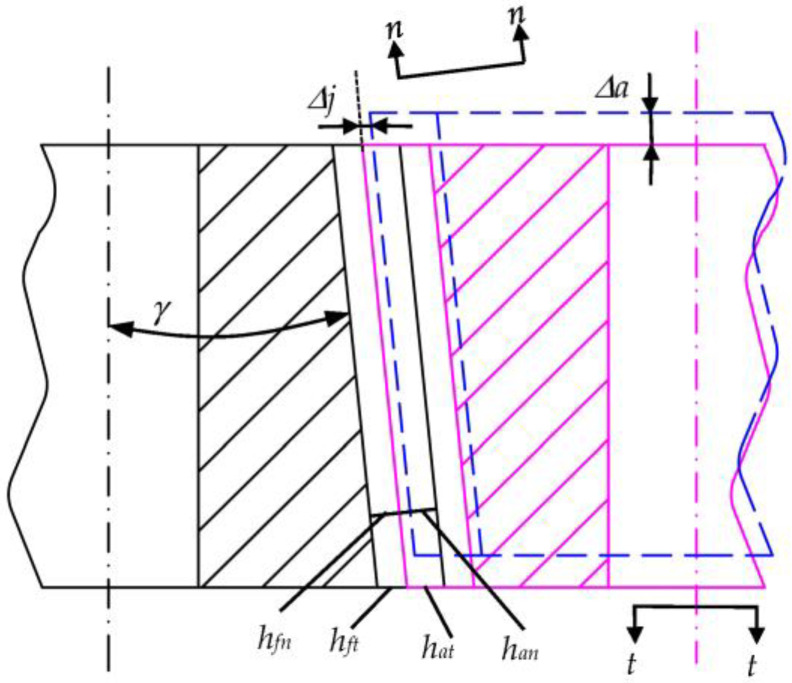
Schematic diagram of parallel-shaft beveloid non-circular gear transmission.

Figure 7 shows a profile chart of a parallel-shaft beveloid non-circular gear drive when the roll angle is *γ*. In Figure 7, *t*-*t* is the end section of the gear, perpendicular to the axis of the gear; *n*-*n* is the normal section of the gear with an angle *γ* to the end section; *h_at_*, *h_ft_*, *h_an,_* and *h_fn_* are the addendum and dedendum of the end section of the gear and the addendum and dedendum of the normal section, respectively. According to the relationship between side length and angle,

(15) $$h_{an}=h_{at}\cos\gamma =h_{at}^{*}m_{t}\cos\gamma =h_{an}^{*}m_{n}$$

According to Equation (14), the relationship between the end section addendum coefficient of parallel-shaft beveloid non-circular gear and the normal section dedendum coefficient is as follows:

(16) $$h_{at}^{*}=h_{an}^{*}\frac{\cos\beta }{\cos\gamma }$$

Similarly, the equation to find the end section tip clearance coefficient C_t_* and tip clearance coefficient C* is Equation (17):

(17) $$C_{t}^{*}=C^{*}\frac{\cos\beta }{\cos\gamma }$$

As mentioned above, the meshing backlash can be reduced by adjusting the axial position of the parallel-shaft beveloid non-circular gear. In Figure 7, when the axial clearance adjustment amount of the parallel-shaft beveloid non-circular gear is set as Δ*a*, the corresponding radial clearance variation Δ*j* of the beveloid non-circular gear is as shown in Equation (18):

(18) Δj=Δa⋅tanγ

According to Equation (18), the clearance variation of parallel-shaft beveloid non-circular gears is related to the axial displacement Δ*a* and rack tool inclination angle *γ*. When the roll angle *γ* = 0°—that is, the rack cutter is a standard straight tooth—the backlash variation is always 0 and has nothing to do with the axial displacement variation of the gear.

Due to the limitations of processing beveloid non-circular gears, this paper only proposes the theoretical model of backlash-eliminating beveloid non-circular gears.

## 5. Simulation and Test

It can be seen from Equations (9) and (10) that the pitch curve shape of the driving and driven non-circular gears is directly related to the length *B* and width *d* of the T-shaped and cross-shaped components. This paper analyzes the influence of the variation in component length and width on the pitch curve shape of non-circular gears on the premise that the other parameters are set. The initial design parameters are shown in Table 1 and Table 2.

### 5.1. Non-Circular Gear Pitch Curve Calculation

**Figure 8:**
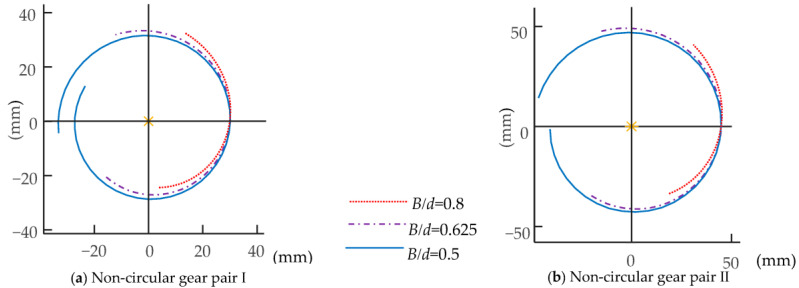
The pitch curve models of non-circular gear pairs I and II.

For the initial design parameters given in Table 1 and Table 2, the two-DOF multi-joint bending mechanism is set. The effective driving angle *θ*_1_ between the joints of each unit of the first bending module is −20° ≤ *θ*_1_ ≤ 20°; that of the second bending module (*θ*_2_) is −25° ≤ *θ*_2_ ≤ 25°. Figure 8 shows the pitch curve model of non-circular gear pairs I and II.

According to Figure 8, when the component aspect ratio *B*/*d* = 0.8, the pitch curve driving angle of the non-circular gear is too small, which makes it impossible to make full use of the non-circular gear effectively. When the aspect ratio of the member is *B*/*d* = 0.5, the rotation angle of the non-circular gear’s pitch curve is more than 360°. The interference phenomenon will occur in the process of the non-circular gear’s movement. When *B*/*d* = 0.625, non-circular gears can be utilized to a greater extent, and interference phenomena can be avoided in the pitch curve. Therefore, this group of parameters can be selected.

**Figure 9:**
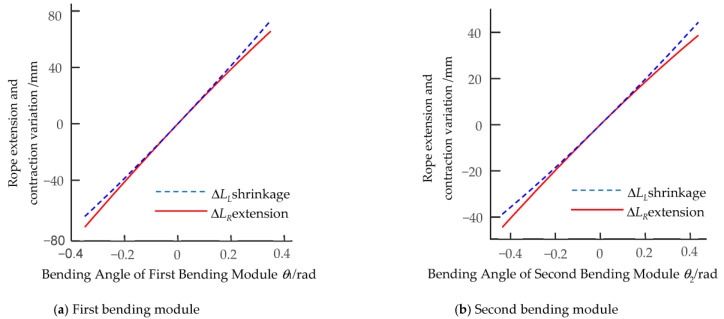
The first and second bending modules’ cable extension and contraction changes.

**Figure 10:**
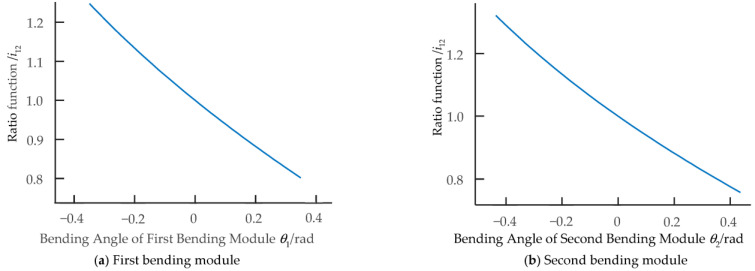
The first and second bending modules’ transmission ratio functions.

For the geometric parameters of the component when the aspect ratio is 0.625, Equations (3) and (5) give the variation of cable extension and contraction and the transmission ratio function of the simple bending mechanism with the single DOF. In order to verify the nonlinear extension and contraction characteristics of the cable, for the initial design parameters given in Table 1 and Table 2, Figure 9 and Figure 10 show the graphs of the extension and contraction changes and the transmission ratio functions of the cable corresponding to the first and second bending modules as the angle of the two-DOF multi-joint bending mechanism changes.

The two-DOF multi-joint bending mechanism is set. The effective driving angle *θ*_1_ between the joints of each unit of the first bending module is −20° ≤ *θ*_1_ ≤ 20°; that of the second bending module (*θ*_2_) is −25° ≤ *θ*_2_ ≤ 25°. Figure 9 shows that the extension and contraction of the left and right cables controlling the bending movement of the biomimetic mechanism undergo nonlinear changes; that is, Δ*L_L_ ≠* Δ*L_R_*. As shown in Figure 10, its transmission ratio function *i*_12_ is a smooth curve when the mechanism is in bending motion. The traditional circular gear can only transmit fixed transmission ratio motion, which cannot meet the design requirements, so the nonlinear transmission characteristics of the non-circular gear are used to drive the cable’s extension and contraction changes.

**Figure 11:**
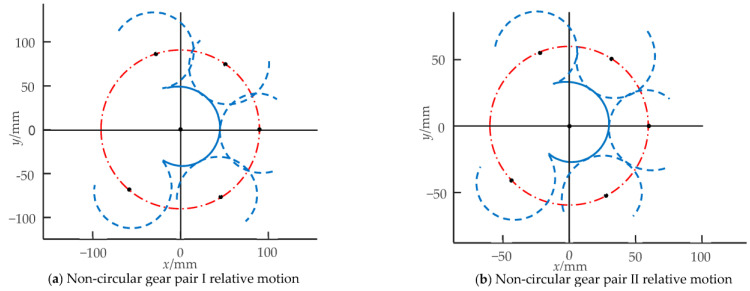
Curve meshing diagrams of non-circular gear pairs.

Based on the pitch curve equations of the driving and driven non-circular gears in Equations (9) and (10), along with the initial design parameters in Table 1 and Table 2, the pitch curve images of two non-circular gear pairs were obtained. Several positions were selected to draw the relative motion images of the non-circular gear pairs, as shown in Figure 11. In the two figures, the solid line on the left is the driving non-circular gear of non-circular gear pairs I and II, and the rest is the corresponding driven non-circular gear. The center distance of non-circular gear pairs I and II is fixed value (*C* = 90 mm, 60 mm). The effective driving angle between the joints of the first bending module is −20° ≤ *θ*_1_ ≤ 20°, and the rotation range of the non-circular gear pair I is −119.9° ≤ *θ_L_*_1_ ≤ 107.5°. The effective driving angle between the joints of the second bending module is −25° ≤ *θ*_1_ ≤ 25°. The rotation range of the corresponding non-circular gear pair II is −127.2° ≤ *θ_L_*_2_ ≤ 110.9°. The effective driving angles of the two non-circular gear pairs are greater than 180°, and there is no interference in the driving rotation range.

### 5.2. Meshing Characteristics of the Tooth Surface without Installation Errors

**Figure 12:**
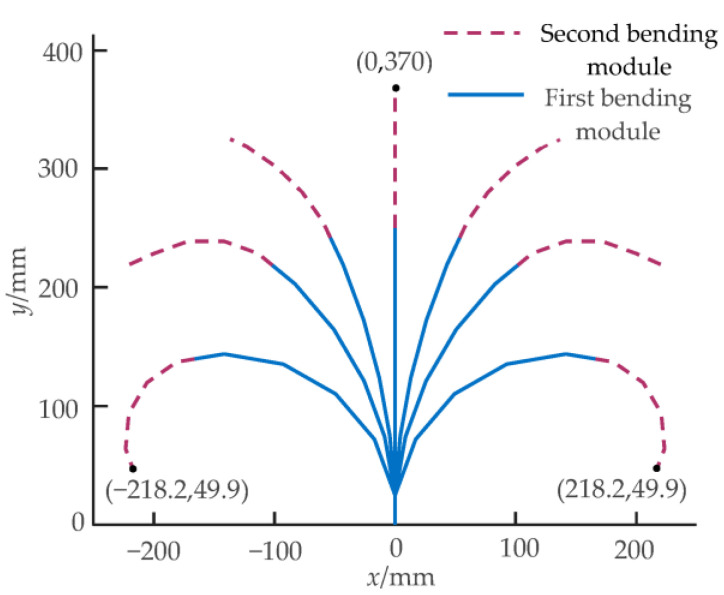
Bionic mechanism center skeleton curve.

When the initial design parameters have been determined, the motion state of the two-DOF multi-joint bionic mechanism and the working space position that the mechanism can achieve depend on the relative angles *θ*_1_ and *θ*_2_ between joints under a single module. According to the above, the effective driving angles between the joints of the first and second bending module units are −20° ≤ *θ*_1_ ≤ 20° and −25° ≤ *θ*_2_ ≤ 25°, respectively. As shown in Figure 12, seven groups of data were selected to draw the workspace state of the central skeleton of the two-DOF multi-joint bionic mechanism as the unit joint angle changes.

For the seven groups of data selected from left to right in Figure 12, the rotation angles between the joints of the first and second bending module units are *θ*_1_ = 20°, 10°, 5°, 0°, −5°, −10°, and −20°, respectively, and *θ*_2_ = 25°, 20°, 10°, 0°, −10°, −20°, and −25°, respectively (taking counterclockwise rotation as positive). When the biomimetic mechanism is in the initial state—that is, *θ*_1_ = *θ*_2_ = 0°—the position coordinate of the center skeleton of the second bending module is (0, 370), which is the farthest distance that the center skeleton of the biomimetic mechanism can extend. The leftmost and rightmost curves are the limit torsion positions that the central skeleton of the mechanism can reach. As shown in Figure 12, the central skeleton’s head coordinates are (−218.2, 49.9), (218.2, 49.9).

**Figure 13:**
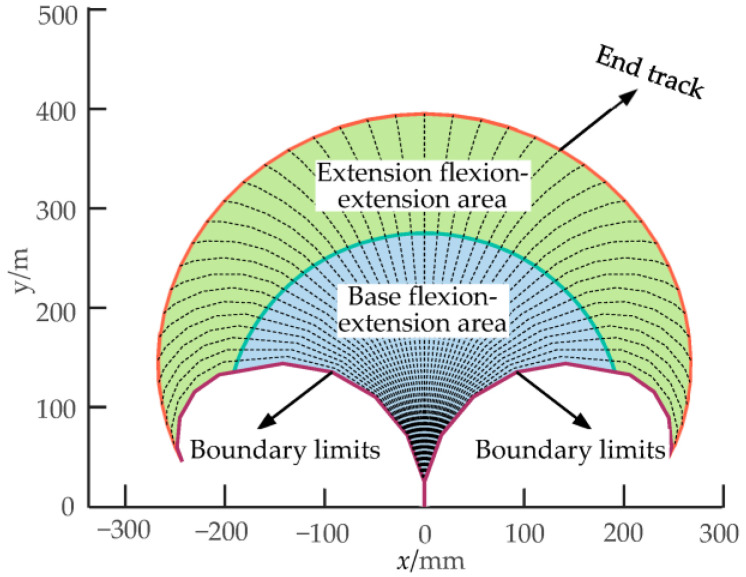
Workspace of the two-DOF multi-joint bionic mechanism.

The flexion and extension area that the central skeleton can sweep during movement is defined as its working space. The area swept by the center skeleton of the first bending module is defined as the base flexion–extension area. The area swept by the center skeleton of the second bending module is defined as the extension–flexion–extension area. The limit position that the central skeleton can reach when bending to both sides is the boundary limit of the bionic mechanism, as shown in Figure 13.

### 5.3. Workspace Comparison Test of Prototype

**Figure 14:**
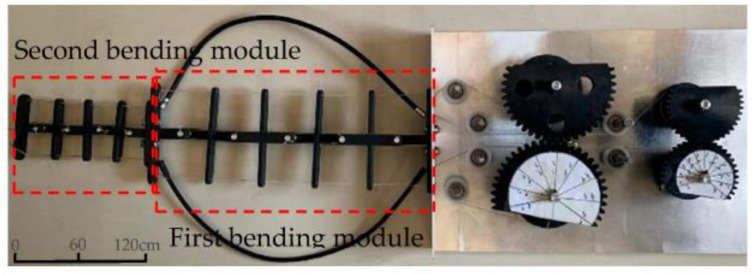
Principle prototype of the two-DOF bionic mechanism.

According to the design scheme proposed in this paper, a two-DOF multi-joint bionic mechanism prototype was manufactured, as shown in Figure 14. In the test, the centerline accuracy of the skeleton of the prototype was tested by changing the driving rotation angle of the non-circular gears and then controlling the extension and contraction of the cables. The test was divided into two working conditions. The rotation angles of the non-circular gear pairs I and II and the bending angles of the corresponding joints of each module unit under the two working conditions are shown in Table 3.

The T-shaped components and cross-shaped components were both 3D printed and made of resin. Resin is a universal material for 3D printing, with a tendency to flow at high temperatures and good stability and wear resistance at room temperature.

Marking points were arranged at the center of the end of the prototype’s first and second bending modules. The position coordinates of the marking points during the bending process of the prototype were recorded and compared with the theoretical spatial position of the bionic mechanism.

**Figure 15:**
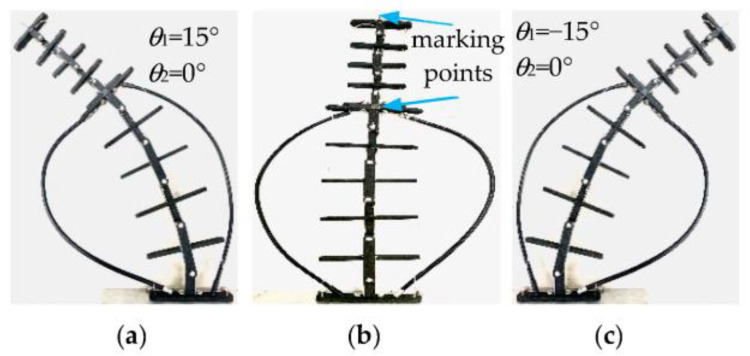
Bending diagram of the prototype under working condition I.

**Figure 16:**
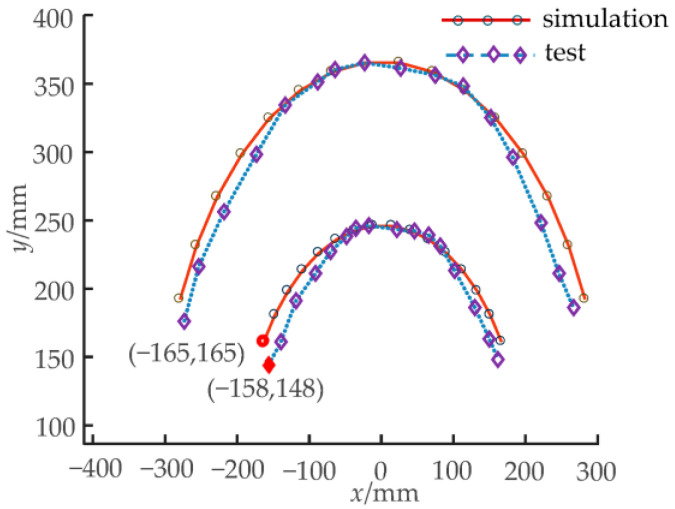
Simulation and test trajectory of the bionic mechanism under working condition I.

Working condition I: The driving rotation angle of non-circular gear pair I is −88.9~81.9°, and non-circular gear pair II remains stationary. At this time, the corresponding first bending module has a theoretical bending swing angle of −15~15°, and the second bending module of the unit joints does not bend. Figure 15 shows the bending changes of the prototype when the rotation angles of each unit joint of the first bending module are −15°, 0°, and 15°, respectively. Figure 16 compares the actual trajectory of the marker points with the theoretical simulation.

**Figure 17:**
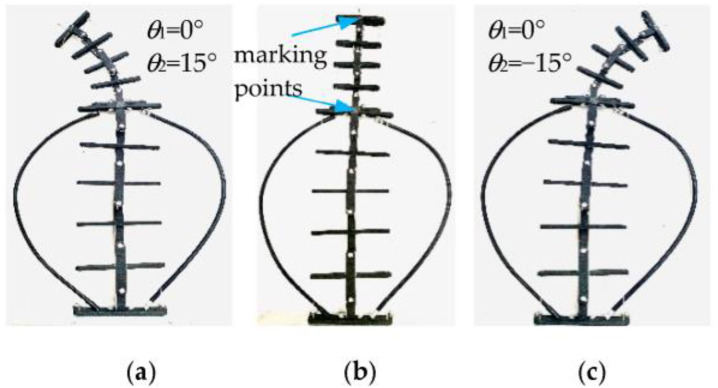
Bending diagram of the prototype under working condition II.

**Figure 18:**
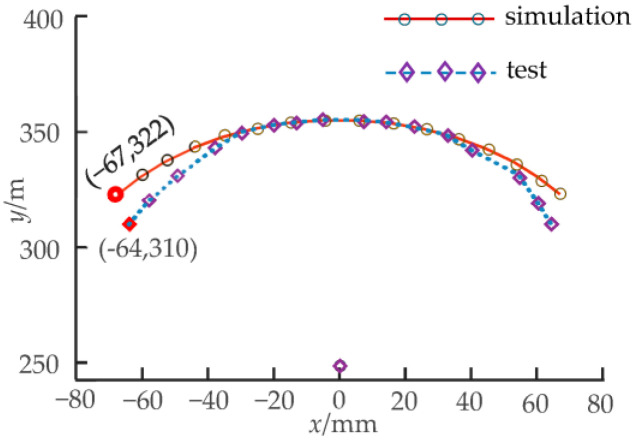
Simulation and test trajectory of the bionic mechanism under working condition II.

Working condition I: The non-circular gear pair I was stationary. The driving rotation angle of the non-circular gear pair II was −74.7~68.9°. At this time, the first bending module unit joints do not bend. The second bending module’s unit joints have a theoretical bending angle of −15~15°. Figure 17 shows the bending changes of the prototype when the rotation angles of each unit joint of the first bending module are −15°, 0°, and 15°, respectively. Figure 18 compares the actual trajectory of the marker points with the theoretical simulation.

As shown in Figure 15, the second bending module is always upright without angular deflection when the first bending module bends in motion. Figure 17 shows the second bending module’s bending movement, where the first bending module is always at rest, so the first and second bending modules can achieve independent movement. The above phenomena can verify the feasibility of the two-DOF decoupling scheme. In the initial bending stage of the bionic mechanism, the simulation trajectory of the marking point is essentially consistent with the test trajectory, and the error between them is small; as the bending angle increases, the error also increases. Under working condition I, when the bending angle of the first bending module is 15°, the simulation and test coordinate points of the marking points are as shown in Figure 16, and the relative error is 7.16%. In Figure 18, the maximum relative error under working condition II is 3.74%. The experimental results show that the experimental test and the simulation calculation are essentially consistent in the bending degree and the changing trend of the bionic mechanism, which can verify the correctness of the theoretical calculation model.

The sources of errors generated during the test are mainly as follows:

(1) The stiffness of the torsion spring used in the prototype is too small to provide enough bending stiffness, so there is a certain error in the assumption of equal curvature of each joint in the bending process.
(2) Due to the error of the initial position of the T-/cross-shaped component and the torsion spring, the hinge points of the multi-joint mechanism are not kept in a straight line at the initial position.

## 6. Conclusions

(1) By designing the pitch curve of the non-circular gear matching the number of joints, the motion control of the multi-joint mechanism can be achieved by using only one motor, without detecting the angle of the cable wheel. This scheme can minimize the control difficulty of multi-joint mechanisms and reduce the system cost.
(2) In order to solve the coupling problem of a two-DOF or multi-DOF multi-joint bending mechanism, a decoupling scheme driven by a Bowden line was proposed. This scheme can achieve the independent control of different bending modules of the bionic mechanism and improve the working space and flexibility of the bionic mechanism.
(3) For high-precision multi-joint mechanisms, the backlash can be reduced by the relative axial movement of beveloid non-circular gears. The influence of hysteresis on the motion accuracy of multi-joint mechanisms can be eliminated.
(4) The non-circular gear-driven multi-joint mechanism can provide a reliable and simple bionic spine for bionic robotic fish, bionic quadruped robots, etc. At the same time, the drive scheme based on non-circular gears can also be extended to cable-driven continuous mechanisms.

## Figures and Tables

**Table 1:** Initial parameters of the first bending module mechanism.

First bending module mechanism	Cable wheel radius, *r* (mm)	35		
	Length of T-shaped component, *B* (mm)	16	25	30
	Length of cross-shaped component, 2*B* (mm)	32	50	60
	Width of cross-shaped component, 2*d* (mm)	40	80	120
	Component length ratio, *B*/*d*	0.8	0.625	0.5
	Total number of components, *N*	5		
	Center distance of non-circular gear, *C* (mm)	90		

**Table 2:** Initial parameters of the second bending module mechanism.

Second bending module mechanism	Cable wheel radius, *r* (mm)	20		
	Length of T-shaped component, *B* (mm)	12	15	20
	Length of cross-shaped component, 2*B* (mm)	24	30	40
	Width of cross-shaped component, 2*d* (mm)	30	60	80
	Component length ratio, *B*/*d*	0.8	0.625	0.5
	Total number of components, *N*	4		
	Center distance of non-circular gear, *C* (mm)	60		

**Table 3:** Drive angles under different working conditions.

Working Condition	GearIration Angle (°)	GearIIration Angle (°)	First Module Bending Angle	Second Module Bending Angle
I	−88.9~81.9°	0°	−15~15°	0°
II	0°	−74.7~68.9°	0°	−15~15°
